# Validation of the prognostic models in acute-on-chronic liver failure precipitated by hepatic and extrahepatic insults

**DOI:** 10.1371/journal.pone.0219516

**Published:** 2019-07-10

**Authors:** Kotchakon Maipang, Pichanun Potranun, Siwaporn Chainuvati, Supot Nimanong, Watcharasak Chotiyaputta, Tawesak Tanwandee, Phunchai Charatcharoenwitthaya

**Affiliations:** Department of Medicine, Division of Gastroenterology, Faculty of Medicine, Siriraj Hospital, Mahidol University, Bangkok, Thailand; Cliniques Universitaires Saint-Luc, BELGIUM

## Abstract

**Background:**

Patients with acute-on-chronic liver failure (ACLF) precipitated by hepatic injury and extrahepatic insults had distinct clinical phenotypes, and prognosis. This study aimed to validate prognostic models for ACLF and to explore their discriminative abilities in ACLF population categorized by the etiologies of precipitating events.

**Methods:**

This study collected data from 343 consecutive cirrhotic patients hospitalized with the diagnosis of ACLF according to the EASL-CLIF-Consortium definition. The discrimination abilities of prognostic models at the onset of ACLF were tested with the concordance index and area under the receiver operating characteristic curve.

**Results:**

Among the entire cohort, 103 patients survived with medical management, nine patients were transplanted, and 231 patients died without liver transplantation. The predictive accuracy of the Chronic Liver Failure-Sequential Organ Failure Assessment (CLIF-SOFA) for 28-day mortality was similar to the CLIF Consortium Organ Failure (CLIF-C OF) but significantly higher than the CLIF Consortium ACLF, the Child-Turcotte-Pugh, the model for end-stage liver disease (MELD), the MELD-sodium, the integrated MELD, and the Acute Physiology and Chronic Health Evaluation II. Of note, 44 patients had acute hepatic insult triggering ACLF (hepatic-ACLF), 244 were exclusively precipitated by bacterial infection or gastrointestinal bleeding (extrahepatic-ACLF), and 55 cases had no any identifiable potential precipitating events. Patients with hepatic-ACLF had significantly higher 28-day mortality than extrahepatic-ACLF patients. The CLIF-SOFA and CLIF-C OF displayed the highest accuracy significantly outperforming other scoring systems in predicting mortality among patients with hepatic-ACLF and those with extrahepatic-ACLF.

**Conclusion:**

The CLIF-SOFA and simpler CLIF-C OF are reliable measures of mortality risk in ACLF patients precipitated by either hepatic or extrahepatic insults. Both validated models could be used to stratify the risk of death and improve management of ACLF.

## Introduction

Acute-on-chronic liver failure (ACLF) is a distinct clinical entity characterized by acute deterioration of liver function in patients with the pre-existing chronic liver disease [1]. Worsening of liver function and subsequently of other end-organs occurs rapidly and follows a precipitating event that directly or indirectly affects hepatocytes [1, 2]. ACLF in patients with acute decompensation is associated with increased short-term mortality due to multisystem organ failure [3–5]. This evidence suggests that patients with ACLF should be recognized early and treated as potentially high-risk patients requiring closer monitoring and interventions to prevent progression to death.

Recently, the European Association for the Study of the Liver-Chronic Liver Failure (EASL-CLIF) Consortium proposed diagnostic criteria for ACLF as defined by the Chronic Liver Failure-Sequential Organ Failure (CLIF-SOFA) score [6]. The CLIF-SOFA scoring system was also formulated to classify ACLF patients into three grades and predict mortality of ACLF patients by addressing organ failures [6–9]. Subsequently, the CLIF Consortium Organ Function (CLIF-C OF) and the CLIF Consortium ACLF (CLIF-C ACLF) were developed to improve the accuracy for the short-term prognosis of ACLF patients [10]. Several studies showed that the organ failure scoring systems were more accurate in predicting mortality of ACLF patients than conventional scoring systems such as Child-Turcotte-Pugh (CTP), the model for end-stage liver disease (MELD), and their variants [10–12]. However, the prognostic value of the models for patients with ACLF needs to be validated further through independent groups of patients with the different etiologies of acute and chronic components of ACLF. Notably, risk stratification with the prognostic scoring systems should help differentiate the group of patients having a high risk of dying shortly after the diagnosis of ACLF from the group of patients at lower risk for complications and mortality.

The objectives of this study were to validate externally different prognostic scores for ACLF patients and to explore their discriminative abilities for the prediction of mortality in ACLF population categorized by the etiologies of precipitating events. This study also sought to determine the clinical application of the reliable prognostic score for early identification of ACLF patients at low and high risk for death.

## Materials and methods

### Study populations

This retrospective cohort study was performed in cirrhotic patients hospitalized with acute decompensation in our institute between 2002 and 2013. Patients were included if they met the following criteria: (a) patient ≥18 years of age; (b) the presence of cirrhosis as determined from clinical, biochemical, radiologic, endoscopic or histopathologic results; (c) acute decompensation of cirrhosis as defined by the acute development of large ascites, hepatic encephalopathy, gastrointestinal hemorrhage, bacterial infection, or any combination. The following patients were excluded: those with human immunodeficiency virus infection, and those who had hepatocellular carcinoma outside Milan criteria or advanced stage cancer, and those who were admitted for a scheduled procedure or treatment. For patients who were readmitted, we only recorded the clinical condition for the first time of admission to avoid double weighing the same patient. The study protocol conformed to the ethical guidelines of the 1975 Helsinki Declaration and was approved by the Siriraj Institutional Review Board with a waiver of patient consent.

ACLF was diagnosed according to the EASL-CLIF Consortium definition [6], which identified organ failures of the liver, coagulation, kidney, circulation, lung, and cerebral systems. Briefly, the definition of organ failure was derived from the CANONIC study [6] (liver failure: serum bilirubin level ≥12 mg/dL; kidney failure: serum creatinine level of ≥2.0 mg/dL or need of renal replacement therapy; cerebral failure: grade III or IV hepatic encephalopathy as per West Haven classification [13]; coagulation failure: international normalized ratio (INR) >2.5 or platelet count ≤20x10^9^/L; circulatory failure: mean arterial pressure <70 mmHg despite adequate fluid resuscitation and need for vasoactive agents; and respiratory failure: PaO_2_/FiO_2_ ≤ 200 or SpO_2_/FiO_2_ ≤214 or need for mechanical ventilation). Patients who had more than two organ failures, single kidney failure, or one organ failure with the presence of kidney dysfunction (creatinine 1.5 to 1.9 mg/dL) and/or mild-to-moderate hepatic encephalopathy were diagnosed as ACLF. The ACLF patients were divided into three groups (ACLF grade 1, ACLF grade 2, and ACLF grade 3) according to the EASL-CLIF Consortium definition [6].

### Data collection

The clinical and laboratory information was collected including age, sex, etiology of cirrhosis, comorbidity, previous episodes of hepatic decompensation, precipitating events, laboratory parameters, events of organ failures, liver transplantation, and causes of death. The event that leads to developing acute decompensation of cirrhosis was defined as a precipitating event of ACLF. The precipitating event was considered as a hepatic insult if the event directly affects hepatocytes such as active alcohol consumption leading to alcoholic hepatitis, hepatitis A or E superimposed infection, acute exacerbation of hepatitis B virus, a flare-up of autoimmune hepatitis, or drug-induced liver injury, and as an extrahepatic insult if there was bacterial infection or upper gastrointestinal bleeding with secondarily affecting the liver. All the variables for computing all models were collected at the onset of ACLF. Prognostic models used in predicting the time-dependent death of ACLF patients included: the Acute Physiology and Chronic Health Evaluation (APACHE)-II, CTP, MELD, MELD-sodium (MELD-Na), integrated MELD (iMELD), CLIF-SOFA, CLIF-C OF, and CLIF-C ACLF scores [7, 10–12]. The main study outcomes included all-cause mortality at 28 days, 90 days, 6 months, and 1 year after admission. Patients were followed until death, liver transplantation, or the last visit.

The APACHE II score (range, 0–71) was calculated on 12 physiologic variables, age, and underlying health. The CTP score (range, 5–15) is measured by hepatic encephalopathy, ascites, albumin, serum bilirubin, and INR. The MELD score (range, 6–40) was calculated as follows: 9.6 × log(creatinine [mg/dL]) + 3.8 × log(bilirubin [mg/dL]) + 11.2 × log(INR) + 6.43. MELD-Na score is modified based on the MELD score and calculated as follows: MELD–Na–[0.025 × MELD × (140 –Na)] + 140. The iMELD model for ACLF patients was calculated as follows: 0.030 × age + 1.759 × (hepatic encephalopathy score) + 0.104 × MELD. The CLIF-SOFA score (range, 0–24) is calculated by the sum of scores for six organ systems, including liver, coagulation, respiratory, cardiovascular, renal, and nervous systems. The CLIF-C OF score (range, 6–18) comprises the modified six organ systems of the CLIF-SOFA score. The CLIF-C ACLF score is modified based on the CLIF-SOFA and calculated as follows: 10 × [0.33 × CLIF-C OF + 0.04 × age + 0.63 × log(white-cell count)– 2].

### Statistical analysis

Continuous variables were summarized as mean ± standard deviation and categorical variables as percentage. Statistical analyses were performed using χ^2^ test or Fisher’s exact test for categorical variables. Student t-test or one-way analysis of variance was used for group comparisons of quantitative data. Cumulative-incidence functions of all-cause mortality in ACLF subgroups were calculated with accounting for the competing risk of liver transplant. Prognostic factors for mortality in ACLF patients were analyzed by Cox regression analysis. Variables found to be associated with mortality on univariate analysis at a probability threshold of <0.10 were included in multivariate analysis with stepwise variable selection. To avoid the effect of collinearity, all prognostic scores and their indexes were not included in the same multivariate models. Variables were expressed as hazard ratio (HR) and 95% confidence interval (CI).

Harrell’s concordance index (C-index) was used to assess the score discrimination ability [14, 15]. C-index values and the corresponding 95% CI were estimated treating the transplanted patients as censored at the end of the follow-up, assuming that none of them could die before [16]. Statistical comparisons of C-index between the prognostic scores were carried out for the main study time-points using the Integrated Discriminating Improvement statistic. A confirmatory analysis was performed to evaluate the discrimination ability of the scoring systems by estimating the area under the receiver operating characteristic curve (AUROC) for each time point, treating transplanted patients as death at the end of the period. Pairwise comparison of the AUROC was done by the Delong test. The sensitivity, specificity, positive predictive value (PPV) and negative predictive value (NPV) were calculated for each cut-off value. All statistical testing was done at the two-tailed α level of 0.05. The SPSS software package version 18.0 (SPSS Inc., Chicago, IL) was used for all analysis.

## Results

### Characteristics of the study population

In 706 consecutive cirrhotic patients hospitalized with acute decompensation, 294 patients presented with ACLF on admission, and 49 patients developed ACLF during their hospital stay according to the EASL-CLIF Consortium definition. Overall, ACLF accounted for 49% of patients admitted to our hospital.

Table 1 displays the patient characteristics and laboratory results of the ACLF cohort. The etiologies of cirrhosis were mainly hepatitis B (38.2%) or hepatitis C (21%), followed by cryptogenic (20.4%), nonalcoholic fatty liver disease (9.3%) and alcoholism (5.0%). The ACLF cohort comprised of a large number of critically ill patients, resembled by a high mean MELD score of 26.8±8.7 and a large proportion of patients categorized as ACLF grades 2 or 3 (73%). In all grades of ACLF, kidney failure was the most common organ failure; however, only 24 of our cohort (9.4%) were initiated on renal replacement therapy. Of note, precipitating events triggering the development of ACLF were identified in 288 patients, and the trigger of the syndrome was not recognized in the remaining 55 patients (16%). The patients with certain precipitating events were categorized into two groups according to types of acute insults: 44 patients precipitated by hepatic insults (hepatic-ACLF) and 244 cases precipitated exclusively by extrahepatic insults (extrahepatic-ACLF). Among hepatic insults, active alcoholism (9.7%) was the most common, followed by acute exacerbation or flare-up of hepatitis B virus (2.0%), hepatotoxic drugs (0.9%), a flare-up of autoimmune hepatitis (0.9%), and superimposed hepatitis E virus infection (0.3%). Bacterial infections were major extrahepatic insults of ACLF in 53.6%, and gastrointestinal hemorrhage was considered as a precipitating factor leading to extrahepatic-ACLF in 22.7%.

**Table 1:** Clinical characteristic, laboratory, and outcomes of patients with acute-on-chronic liver failure.

Variables	Total ACLF (n = 343)	Hepatic-ACLF (n = 44)	Extrahepatic-ACLF (n = 244)	*P* Value*
**Age, years**	60.5±13.8	54.7±11.9	61.3±13.5	0.003
**Male, no. (%)**	207 (60.4)	36 (81.2)	139 (57.0)	0.002
**Previous decompensation, no. (%)**				
Total	231 (67.4)	32 (72.7)	158 (64.8)	0.304
Ascites	113 (32.9)	20 (45.5)	76 (31.2)	0.064
Hepatic encephalopathy	27 (7.9)	2 (4.6)	17 (7.0)	0.748
Variceal hemorrhage	91 (26.5)	11 (25.0)	67 (27.5)	0.736
Spontaneous bacterial peritonitis	76 (22.2)	17 (38.6)	46 (18.9)	0.004
**Etiology of cirrhosis, no. (%)**				
Hepatitis B virus	131 (38.2)	20 (45.5)	90 (36.9)	0.282
Hepatitis C virus	72 (21.0)	8 (18.2)	54 (22.1)	0.557
Alcohol	17 (5.0)	10 (22.7)	7 (2.9)	<0.001
Autoimmune hepatitis	5 (1.5)	1 (2.3)	4 (1.6)	0.566
Nonalcoholic fatty liver disease	32 (9.3)	0	26 (10.7)	0.019
Cryptogenic	70 (20.4)	3 (6.8)	51 (20.9)	0.028
Others	16 (4.7%)	2 (4.5)	12 (4.9)	1.000
**Organ failure, no. (%)**				
Liver	118 (34.4)	25 (56.8)	72 (29.5)	<0.001
Kidney	256 (74.6)	33 (75.0)	182 (74.6)	0.954
Cerebral	131 (38.2)	19 (43.2)	80 (32.8)	0.182
Coagulation	113 (32.9)	23 (52.3)	75 (30.7)	0.006
Circulation	197 (57.4)	30 (68.2)	140 (57.4)	0.180
Respiratory	86 (25.1)	16 (36.4)	51 (20.9)	0.026
**Laboratory parameters**				
Hemoglobin, g/dL	9.7±2.1	9.8±2.4	9.6±2.1	0.635
Leukocyte count, cells/mL	11,232±7,100	12,099±6,790	11,375±7,107	0.532
Platelet count, x10^9^/L	121±87	137±98	122±86	0.281
International normalized ratio	2.02±0.97	2.32±1.19	1.99±0.94	0.042
Total bilirubin, mg/dL	9.5±10.5	14.4±11.8	8.2±9.1	0.002
Albumin, g/dL	2.4±0.6	2.4±0.6	2.4±0.6	0.730
Alanine aminotransferase, U/L	73±129	100±95	69±131	0.059
γ-Glutamyltranspeptidase, U/L	141±140	162±193	132±116	0.516
Creatinine, mg/dL	2.44±1.79	2.23±1.60	2.35±1.52	0.638
Sodium, mmol/L	131±7	130±7	132±7	0.202
**Mean arterial pressure, mmHg**	77±18	73±21	78±18	0.799
**Severity score**				
APACHE II	21.7±6.3	21.5±7.6	21.7±6.3	0.899
CTP	11.3±2.4	12.0±2.2	11.2±2.4	0.051
MELD	26.8±8.7	29.7±10.4	26.1±8.2	0.032
MELD-Na	29.1±8.0	30.8±8.3	28.7±8.0	0.128
iMELD	7.4±2.9	7.8±3.0	7.1±2.8	0.089
CLIF-SOFA	13.0±4.6	14.9±4.7	12.6±4.4	0.002
CLIF-C OF	11.7±2.8	13.2±2.9	11.4±2.7	<0.001
CLIF-C ACLF	57.1±10.9	60.1±9.8	56.4±10.7	0.034
**Grade of ACLF, no. (%)**				
Grade 1	93 (27.1)	3 (6.8)	74 (30.3)	<0.001
Grade 2	86 (25.1)	10 (22.7)	69 (28.3)	
Grade 3	164 (47.8)	31 (70.5)	101 (41.4)	
**All-cause mortality, no. (%)**				
28-day	180 (52.5)	29 (65.9)	119 (48.8)	0.036
90-day	215 (62.7)	32 (72.7)	143 (58.6)	0.077
6-month	226 (65.9)	33 (75.0)	153 (62.7)	0.117
1-year	231 (67.4)	33 (75.0)	158 (64.8)	0.186

Data are expressed as mean ± standard deviation or number (%). ACLF, acute-on-chronic liver failure; APACHE II, acute physiology and chronic health evaluation II; CTP, Child-Turcotte-Pugh; CLIF-SOFA, chronic liver failure-sequential organ failure assessment; CLIF-C ACLF, CLIF consortium acute-on-chronic liver failure; CLIF-C OF, CLIF consortium organ function; MELD, model for end-stage liver disease; MELD-Na, model for end-stage liver disease-sodium; iMELD, integrated model for end-stage liver disease.

*p-value for comparison between the hepatic-ACLF group and the extrahepatic-ACLF group.

Clinical characteristics of ACLF patients according to types of acute insults are shown in Table 1. Hepatic-ACLF patients were predominantly male and were younger than those with extrahepatic-ACLF. Almost half of the hepatic-ACLF patients had ascites, and most of them had a history of previous hepatic decompensation with spontaneous bacterial peritonitis. Alcoholic cirrhosis was highly prevalent in hepatic-ACLF patients, and their underlying liver disease was more severe as defined by higher MELD score and ACLF grade, whereas those with extrahepatic-ACLF had higher proportions of cryptogenic cirrhosis and nonalcoholic fatty liver disease. Hepatic injury-related parameters, such as serum aminotransferase, total bilirubin, and INR were significantly higher in the hepatic-ACLF group. Concerning organ failures, patients with hepatic-ACLF developed higher frequency of liver and coagulation failures than those with extrahepatic-ACLF. Besides, hepatic-ACLF patients had significantly a higher occurrence of respiratory failure.

### Prognostic factors for mortality in patients with ACLF

One-hundred and three patients (30%) survived with medical management, 231 patients (67.4%) died without liver transplantation, and nine patients (2.6%) underwent deceased donor liver transplantation. Seven of the transplanted patients survived at the end of the study period with a mean follow‐up period of 86 months (range 1‐123 months) after liver transplantation. Two transplant recipients died of sepsis during the same admission as liver transplantation. The 90-day, 1-year, and 5-year survival rates after liver transplantation were 89%, 78%, and 78% respectively.

Of the overall cohort, the 28-day mortality rates in patients with ACLF grade 1 (n = 93), ACLF grade 2 (n = 86) and ACLF grade 3 (n = 164) were 20.4%, 34.9% and 79.9% (p <0.001), respectively and 90-day mortality rates was 36.6%, 43.0% and 87.8% (p<0.001), respectively. The mortality rates at 6 months and 1 year rose as the grade of ACLF increased, reaching 88% in patients with ACLF grade 3. Patients with hepatic ACLF had significantly higher proportions of ACLF grade 2 or 3 compared with extrahepatic-ACLF patients. The hepatic-ACLF group had significantly higher 28-day mortality than the extrahepatic-ACLF group (65.9% vs. 48.8%, p = 0.036), whereas both groups had comparably high 90-day, 6-month, and 1-year mortality (Table 1). Using the cumulative incidence function, there was no significant difference in the risk of mortality during the 1-year period between hepatic-ACLF and extrahepatic-ACLF groups (subdistribution HR 1.51; 95%CI 0.93–2.45, p = 0.098).

In an exploratory analysis, we found that when the hepatic-ACLF group stratified by the presence of extrahepatic organ failure, 42 (95%) of 44 hepatic-ACLF patients developed extrahepatic organ failures and their overall mortalities were 69% at 28 days, 76% at 90 days, and 79% at 6 months and 1 year. Only two ACLF patients precipitated by hepatic insults did not develop extrahepatic organ failure and survived during the study period. The hepatic-ACLF patients with extrahepatic organ failure had significantly higher 28-day mortality than the extrahepatic-ACLF group, whereas both groups had comparable 90-day, 6-month, and 1-year mortality. However, the cumulative incidence function revealed no significant difference in the risk of mortality during the 1-year period between both hepatic and extrahepatic-ACLF groups (subdistribution HR 1.61; 95%CI 0.99–2.62, p = 0.055).

Univariate analysis using Cox proportional hazards model showed that previous episodes of liver decompensation, baseline mean arterial pressure, the use of vasopressive agents and mechanical ventilatory support, a complication of hepatic encephalopathy during hospitalization, and the individual laboratory parameters of each prognostic score were significantly associated with 28-day mortality (Table 2). All the prognostic models (the CLIF-SOFA, CLIF-C OF, CLIF-C ACLF, CTP, MELD, MELD-Na, iMELD, and APACHE II) appear to be significantly predictive of mortality. In the multivariate analysis excluding all scoring systems to avoid collinearity, hepatic encephalopathy during hospitalization (HR, 1.28; 95% CI, 1.14–1.44), the use of vasopressive agents (HR, 2.38; 95% CI, 1.61–3.54), mechanical ventilation (HR, 1.50; 95% CI, 1.08–2.08), and baseline values of INR (HR, 1.30; 95% CI, 1.13–1.50), total bilirubin (HR, 1.02; 95% CI, 1.01–1.03), and albumin (HR, 0.76, 95% CI, 0.58–0.99) had independent prognostic significance for predicting overall mortality.

**Table 2:** Prognostic factors for 28-day mortality in patients with acute-on-chronic liver failure.

Characteristic	Beta coefficient	Standard error	Hazard ratio (95%CI)	*P* Value
**Age, years**	-0.007	0.006	0.993 (0.983–1.004)	0.219
**Male sex**	0.275	0.154	1.316 (0.973–1.782)	0.075
**Etiology of cirrhosis**				
Hepatitis B virus	0.057	0.154	1.059 (0.783–1.432)	0.709
Hepatitis C virus	0.148	0.181	1.159 (0.813–1.653)	0.415
Alcohol	0.103	0.342	1.108 (0.567–2.166)	0.765
Nonalcoholic fatty liver disease	-0.528	0.288	0.590 (0.335–1.038)	0.067
Cryptogenic	-0.043	0.183	0.958 (0.669–1.370)	0.813
**Presence of hepatocellular carcinoma**	0.231	0.158	1.259 (0.925–1.714)	0.145
**Previous liver decompensation**	0.380	0.170	1.463 (1.049–2.040)	0.025
**Complications during admission**				
Ascites	0.044	0.151	1.045 (0.777–1.406)	0.770
Spontaneous bacterial peritonitis	-0.112	0.164	0.894 (0.649–1.232)	0.493
Variceal hemorrhage	0.270	0.158	1.310 (0.962–1.784)	0.087
Hepatic encephalopathy	0.373	0.056	1.452 (1.300–1.622)	<0.001
Bacterial infection	-0.048	0.153	0.953 (0.707–1.285)	0.752
**Mean arterial pressure (mmHg)**	-0.015	0.004	0.985 (0.977–0.994)	<0.001
**Use of vasopressive agent**	1.215	0.180	3.371 (2.373–4.788)	<0.001
**Mechanical ventilation**	0.902	0.151	2.465 (1.838–3.306)	<0.001
**Renal replacement therapy**	-0.221	0.299	0.802 (0.448–1.437)	0.461
**Laboratory data**				
Hemoglobin, g/dL	0.007	0.036	1.007 (0.939–1.079)	0.852
Leukocyte count, 10^3^/mL	0.013	0.010	1.014 (0.995–1.033)	0.168
Platelet count, 10^9^/L	0.001	0.001	1.001 (0.999–1.003)	0.156
International normalized ratio	0.342	0.059	1.407 (1.254–1.580)	<0.001
Total bilirubin, mg/dL	0.026	0.006	1.026 (1.014–1.038)	<0.001
Albumin, g/dL	-0.324	0.129	0.723 (0.562–0.931)	0.012
Alanine aminotransferase, U/L	0.002	<0.001	1.002 (1.001–1.003)	<0.001
γ-Glutamyltranspeptidase, U/L	-0.001	0.001	0.999 (0.997–1.001)	0.435
Creatinine, mg/dL	-0.051	0.045	0.950 (0.870–1.038)	0.260
Sodium, mmol/L	-0.012	0.011	0.988 (0.967–1.008)	0.244
**APACHE II score**	0.073	0.012	1.076 (1.051–1.101)	<0.001
**CTP score**	0.230	0.037	1.259 (1.171–1.352)	<0.001
**MELD score**	0.045	0.008	1.046 (1.030–1.062)	<0.001
**MELD-Na score**	0.050	0.009	1.051 (1.032–1.071)	<0.001
**iMELD score**	0.213	0.028	1.237 (1.170–1.308)	<0.001
**CLIF-SOFA score**	0.168	0.017	1.182 (1.143–1.223)	<0.001
**CLIF-C OF score**	0.246	0.026	1.279 (1.217–1.345)	<0.001
**CLIF-C ACLF score**	0.059	0.007	1.061 (1.046–1.076)	<0.001

APACHE II, acute physiology and chronic health evaluation II; CTP, Child-Turcotte-Pugh; CLIF-SOFA, chronic liver failure-sequential organ failure assessment; CLIF-C ACLF, CLIF consortium acute-on-chronic liver failure; CLIF-C OF, CLIF consortium organ function; MELD, model for end-stage liver disease; MELD-Na, model for end-stage liver disease-sodium; iMELD, integrated model for end-stage liver disease.

### Validation of the prognostic scoring systems in patients with ACLF

Comparisons of C-index between eight scoring systems used to determine the prognosis of ACLF are presented in Table 3. The C-index of CLIF-SOFA for 28-day, 90-day, 6-month, and 1-year mortality (0.84, 0.85, 0.80, and 0.80) was significantly better than those corresponding to other prognostic scores including CTP, MELD, MELD-Na, iMELD, and APACHE II. However, the ability of CLIF-SOFA was comparable with that of CLIF-C OF in predicting 28-day and 6-month mortality (0.83 and 0.78) and that of CLIF-C ACLF in predicting 6-month, and 1-year mortality (0.77 and 0.77).

**Table 3:** The time-dependent concordance index (C-index) of the scoring systems in predicting mortality for acute-on-chronic liver failure.

	28-day mortality		90-day mortality		6-month mortality		1-year mortality	
	C-index (95%CI)	p-value*	C-index (95%CI)	p-value*	C-index (95%CI)	p-value*	C-index (95%CI)	p-value*
**All ACLF patients (n = 343)**								
**CLIF-SOFA**	0.84 (0.80–0.88)		0.85 (0.81–0.89)		0.80 (0.76–0.85)		0.80 (0.76–0.85)	
**CLIF-C OF**	0.83 (0.79–0.87)	0.133	0.82 (0.78–0.86)	0.011	0.78 (0.73–0.83)	0.064	0.78 (0.73–0.83)	0.018
**CLIF-C ACLF**	0.79 (0.74–0.84)	0.007	0.80 (0.75–0.85)	0.014	0.77 (0.72–0.82)	0.139	0.77 (0.72–0.82)	0.113
**CTP**	0.70 (0.64–0.75)	<0.001	0.67 (0.61–0.73)	<0.001	0.64 (0.58–0.70)	<0.001	0.63 (0.57–0.69)	<0.001
**MELD**	0.63 (0.57–0.69)	<0.001	0.60 (0.54–0.66)	<0.001	0.56 (0.50–0.62)	<0.001	0.56 (0.50–0.62)	<0.001
**MELD-Na**	0.63 (0.57–0.69)	<0.001	0.59 (0.53–0.65)	<0.001	0.56 (0.50–0.62)	<0.001	0.56 (0.50–0.62)	<0.001
**iMELD**	0.73 (0.68–0.78)	<0.001	0.71 (0.65–0.76)	<0.001	0.67 (0.62–0.73)	<0.001	0.68 (0.62–0.74)	<0.001
**APACHE II**	0.69 (0.63–0.74)	<0.001	0.65 (0.60–0.71)	<0.001	0.63 (0.57–0.69)	<0.001	0.63 (0.57–0.69)	<0.001
**Hepatic-ACLF patients (n = 44)**								
**CLIF-SOFA**	0.93 (0.85–1.0)		0.97 (0.93–1.0)		0.94 (0.87–1.0)		0.94 (0.87–1.0)	
**CLIF-C OF**	0.92 (0.83–1.0)	0.808	0.97 (0.93–1.0)	0.947	0.94 (0.87–1.0)	0.847	0.94 (0.87–1.0)	0.847
**CLIF-C ACLF**	0.81 (0.66–0.96)	0.027	0.90 (0.80–0.99)	0.122	0.88 (0.78–0.99)	0.282	0.88 (0.78–0.99)	0.282
**CTP**	0.85 (0.74–0.98)	0.240	0.86 (0.73–0.99)	0.133	0.83 (0.68–0.97)	0.124	0.83 (0.68–0.97)	0.124
**MELD**	0.71 (0.55–0.88)	0.014	0.72 (0.55–0.89)	0.006	0.69 (0.51–0.87)	0.008	0.69 (0.51–0.87)	0.008
**MELD-Na**	0.67 (0.50–0.84)	0.004	0.69 (0.51–0.87)	0.003	0.65 (0.47–0.84)	0.003	0.65 (0.47–0.84)	0.003
**iMELD**	0.81 (0.67–0.95)	0.111	0.82 (0.67–0.96)	0.039	0.79 (0.64–0.94)	0.052	0.79 (0.64–0.94)	0.052
**APACHE II**	0.63 (0.44–0.81)	0.004	0.61 (0.40–0.82)	0.001	0.60 (0.37–0.83)	0.004	0.60 (0.37–0.83)	0.004
**Extrahepatic-ACLF patients (n = 244)**								
**CLIF-SOFA**	0.82 (0.77–0.88)		0.82 (0.77–0.87)		0.76 (0.70–0.82)		0.76 (0.70–0.82)	
**CLIF-C OF**	0.81 (0.75–0.86)	0.115	0.80 (0.74–0.85)	0.056	0.75 (0.68–0.81)	0.222	0.74 (0.68–0.80)	0.079
**CLIF-C ACLF**	0.79 (0.74–0.85)	0.185	0.80 (0.74–0.85)	0.421	0.76 (0.70–0.82)	0.928	0.76 (0.70–0.82)	0.992
**CTP**	0.67 (0.61–0.74)	<0.001	0.65 (0.58–0.72)	<0.001	0.61 (0.54–0.68)	<0.001	0.60 (0.52–0.67)	<0.001
**MELD**	0.62 (0.55–0.69)	<0.001	0.58 (0.51–0.65)	<0.001	0.54 (0.47–0.61)	<0.001	0.54 (0.47–0.62)	<0.001
**MELD-Na**	0.61 (0.54–0.69)	<0.001	0.58 (0.51–0.65)	<0.001	0.54 (0.47–0.61)	<0.001	0.54 (0.47–0.62)	<0.001
**iMELD**	0.72 (0.65–0.78)	<0.001	0.70 (0.62–0.76)	<0.001	0.66 (0.59–0.73)	0.002	0.67 (0.60–0.74)	0.003
**APACHE II**	0.73 (0.66–0.79)	0.007	0.69 (0.63–0.76)	0.001	0.67 (0.60–0.74)	0.016	0.67 (0.60–0.74)	0.014

APACHE II, acute physiology and chronic health evaluation II; CTP, Child-Turcotte-Pugh; CLIF-SOFA, chronic liver failure-sequential organ failure assessment; CLIF-C ACLF, CLIF consortium acute-on-chronic liver failure; CLIF-C OF, CLIF consortium organ function; MELD, model for end-stage liver disease; MELD-Na, model for end-stage liver disease-sodium; iMELD, integrated model for end-stage liver disease.

*p-value for comparison with the CLIF-SOFA score.

**Fig 1:**
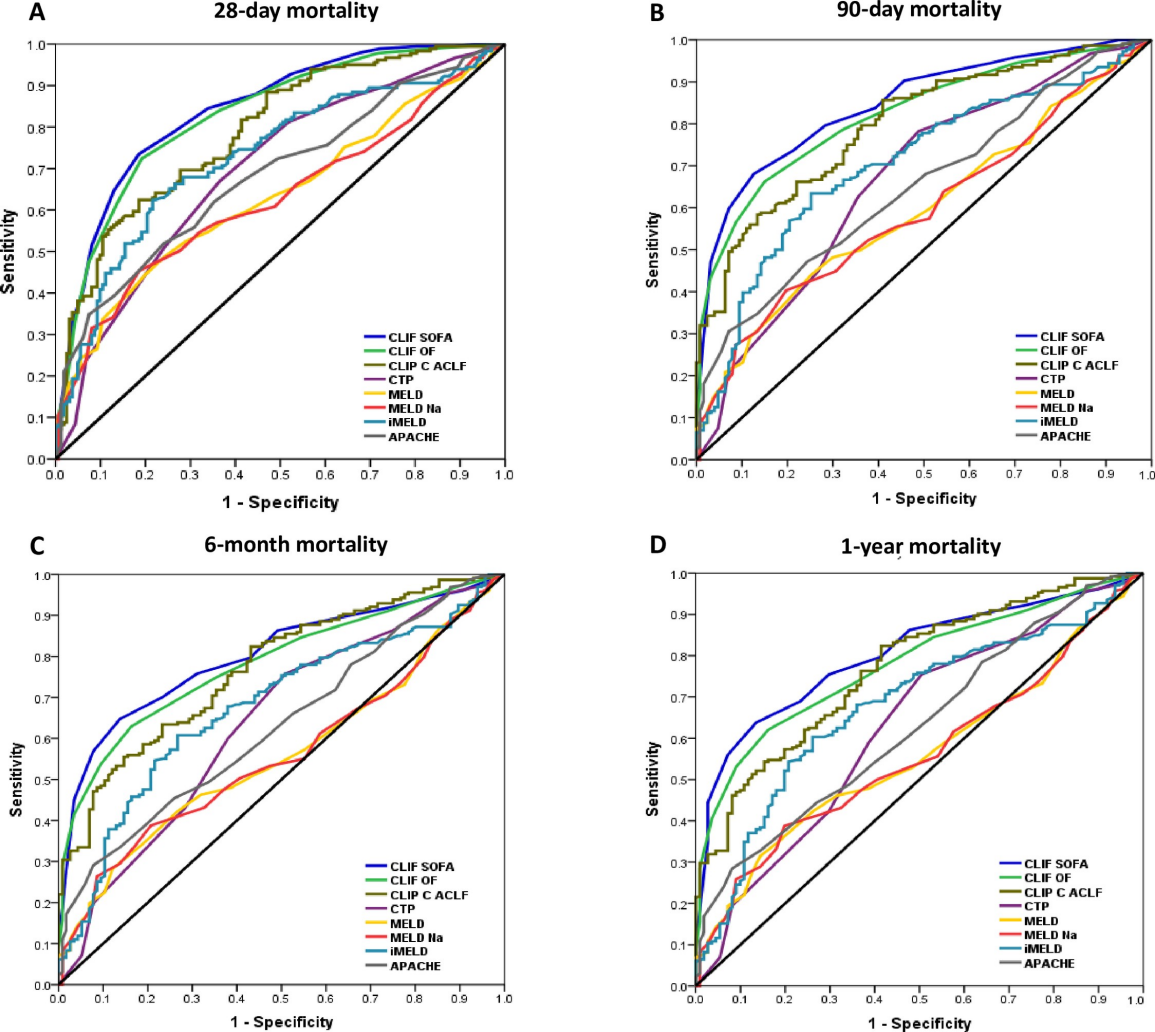
Comparison of the prognostic scoring systems for acute-on-chronic liver failure. The discriminative ability of scoring systems for predicting 28-day (A), 90-day (B), 6-month (C) and 1-year (D) mortalities in acute-on-chronic liver failure.

The further analysis was carried out by comparing the AUROCs corresponding to CLIF-SOFA, CLIF-C OF, CLIF-C ACLF, CTP, MELD, MELD-Na, iMELD, and APACHE II (Table 4). The results confirm the superiority of CLIF-SOFA (AUROC, 0.84; 95% CI, 0.80–0.88) in the predictive ability of 28-day mortality concerning CLIF-C ACLF, CTP, MELD, MELD-Na, iMELD, and APACHE II as shown in Fig 1A and S1 File. Similarly, the CLIF-SOFA score had the best discriminatory power for determining 90-day mortality (AUROC, 0.85; 95% CI, 0.81–0.89), as shown in Figs 1B and S1. However, the ability of CLIF-SOFA was comparable with CLIF-C OF in predicting 28-day and 6-month mortality. Furthermore, the CLIF-SOFA score outperformed other comparator systems except for the CLIF-C ACLF score, displaying the highest AUROCs of 0.80 (95% CI, 0.76–0.85) and 0.81 (95% CI, 0.76–0.85) for predicting 6-month, and 1-year mortality (Figs 1C and 1D and S1), respectively.

**Table 4:** Comparison of the discrimination ability of the scoring systems in predicting mortality of acute-on-chronic liver failure in relation to the types of precipitating events.

	28-day mortality		90-day mortality		6-month mortality		1-year mortality	
	AUROC (95%CI)	p-value*	AUROC (95%CI)	p-value*	AUROC (95%CI)	p-value*	AUROC (95%CI)	p-value*
**All ACLF patients (n = 343)**								
**CLIF-SOFA**	0.84 (0.80–0.88)		0.85 (0.81–0.89)		0.80 (0.76–0.85)		0.81 (0.76–0.85)	
**CLIF-C OF**	0.83 (0.79–0.87)	0.134	0.82 (0.78–0.87)	0.010	0.78 (0.74–0.83)	0.064	0.78 (0.73–0.83)	0.018
**CLIF-C ACLF**	0.79 (0.74–0.83)	0.007	0.80 (0.75–0.84)	0.013	0.77 (0.72–0.82)	0.137	0.77 (0.72–0.82)	0.111
**CTP**	0.70 (0.64–0.75)	<0.001	0.67 (0.61–0.73)	<0.001	0.64 (0.58–0.70)	<0.001	0.63 (0.57–0.69)	<0.001
**MELD**	0.63 (0.57–0.69)	<0.001	0.59 (0.54–0.66)	<0.001	0.56 (0.50–0.62)	<0.001	0.56 (0.50–0.62)	<0.001
**MELD-Na**	0.63 (0.57–0.69)	<0.001	0.60 (0.53–0.65)	<0.001	0.56 (0.50–0.62)	<0.001	0.56 (0.50–0.62)	<0.001
**iMELD**	0.73 (0.68–0.78)	<0.001	0.71 (0.65–0.76)	<0.001	0.68 (0.62–0.73)	<0.001	0.68 (0.62–0.74)	<0.001
**APACHE II**	0.69 (0.63–0.74)	<0.001	0.65 (0.59–0.71)	<0.001	0.63 (0.58–0.69)	<0.001	0.63 (0.57–0.69)	<0.001
**Hepatic-ACLF patients (n = 44)**								
**CLIF-SOFA**	0.93 (0.85–1.0)		0.97 (0.93–1.0)		0.94 (0.87–1.0)		0.94 (0.87–1.0)	
**CLIF-C OF**	0.92 (0.83–1.0)	0.806	0.97 (0.93–1.0)	0.947	0.94 (0.88–1.0)	0.846	0.94 (0.88–1.0)	0.846
**CLIF-C ACLF**	0.81 (0.66–0.95)	0.021	0.90 (0.80–0.99)	0.113	0.88 (0.78–0.98)	0.274	0.88 (0.78–0.98)	0.274
**CTP**	0.85 (0.74–0.96)	0.231	0.86 (0.74–0.99)	0.127	0.83 (0.68–0.97)	0.119	0.83 (0.68–0.97)	0.119
**MELD**	0.71 (0.56–0.87)	0.010	0.72 (0.55–0.89)	0.004	0.69 (0.51–0.87)	0.006	0.69 (0.51–0.87)	0.006
**MELD-Na**	0.67 (0.51–0.84)	0.002	0.69 (0.52–0.87)	0.001	0.65 (0.47–0.84)	0.002	0.65 (0.47–0.84)	0.002
**iMELD**	0.81 (0.68–0.94)	0.102	0.82 (0.68–0.95)	0.033	0.79 (0.64–0.93)	0.046	0.79 (0.64–0.93)	0.046
**APACHE II**	0.63 (0.45–0.81)	0.002	0.61 (0.40–0.82)	<0.001	0.60 (0.37–0.82)	0.002	0.60 (0.37–0.82)	0.002
**Extrahepatic-ACLF patients (n = 244)**								
**CLIF-SOFA**	0.82 (0.77–0.88)		0.82 (0.77–0.87)		0.76 (0.70–0.82)		0.77 (0.71–0.82)	
**CLIF-C OF**	0.81 (0.75–0.86)	0.116	0.80 (0.74–0.85)	0.056	0.74 (0.69–0.81)	0.222	0.74 (0.68–0.80)	0.079
**CLIF-C ACLF**	0.79 (0.74–0.85)	0.184	0.80 (0.75–0.86)	0.416	0.76 (0.70–0.82)	0.933	0.76 (0.70–0.83)	0.986
**CTP**	0.67 (0.61–0.74)	<0.001	0.65 (0.58–0.72)	<0.001	0.61 (0.54–0.68)	<0.001	0.60 (0.53–0.67)	<0.001
**MELD**	0.62 (0.55–0.69)	<0.001	0.58 (0.51–0.65)	<0.001	0.54 (0.47–0.61)	<0.001	0.54 (0.47–0.62)	<0.001
**MELD-Na**	0.61 (0.54–0.69)	<0.001	0.58 (0.51–0.65)	<0.001	0.54 (0.47–0.61)	<0.001	0.54 (0.47–0.62)	<0.001
**iMELD**	0.72 (0.65–0.78)	<0.001	0.70 (0.63–0.76)	<0.001	0.66 (0.60–0.73)	0.002	0.67 (0.60–0.74)	0.003
**APACHE II**	0.72 (0.66–0.79)	0.004	0.69 (0.62–0.75)	<0.001	0.67 (0.60–0.73)	0.012	0.67 (0.60–0.73)	0.011

AUROC, Area under the receiver operating curve; APACHE II, acute physiology and chronic health evaluation II; CTP, Child-Turcotte-Pugh; CLIF-SOFA, chronic liver failure-sequential organ failure assessment; CLIF-C ACLF, CLIF consortium acute-on-chronic liver failure; CLIF-C OF, CLIF consortium organ function; MELD, model for end-stage liver disease; MELD-Na, model for end-stage liver disease-sodium; iMELD, integrated model for end-stage liver disease.

*p-value for comparison with the CLIF-SOFA score.

The performance of the scoring systems was verified within a subgroup of patients with hepatic-ACLF and a subgroup of patients with extrahepatic-ACLF (Tables 3 and 4). The predictive abilities of the CLIF-SOFA for 28-day, 90-day, 6-month, and 1-year mortality were satisfactory with AUROCs of 0.93, 0.97, 0.94, and 0.94 in the hepatic-ACLF subgroup, respectively and AUROCs of 0.82, 0.82, 0.76, and 0.77 in the extrahepatic-ACLF group, respectively. The discriminatory power of the CLIF-SOFA for determining short-term and long-term mortality was comparable with the CLIF-C OF and CTP among the hepatic-ACLF cohort (Table 4). The ability of the CLIF-SOFA was similar to the CLIF-C OF and CLIF-C ACLF for predicting short-term and long-term mortality in the extrahepatic-ACLF cohort.

### Application of the CLIF-SOFA and CLIF-C OF scores in patients with ACLF

**Fig 2:**
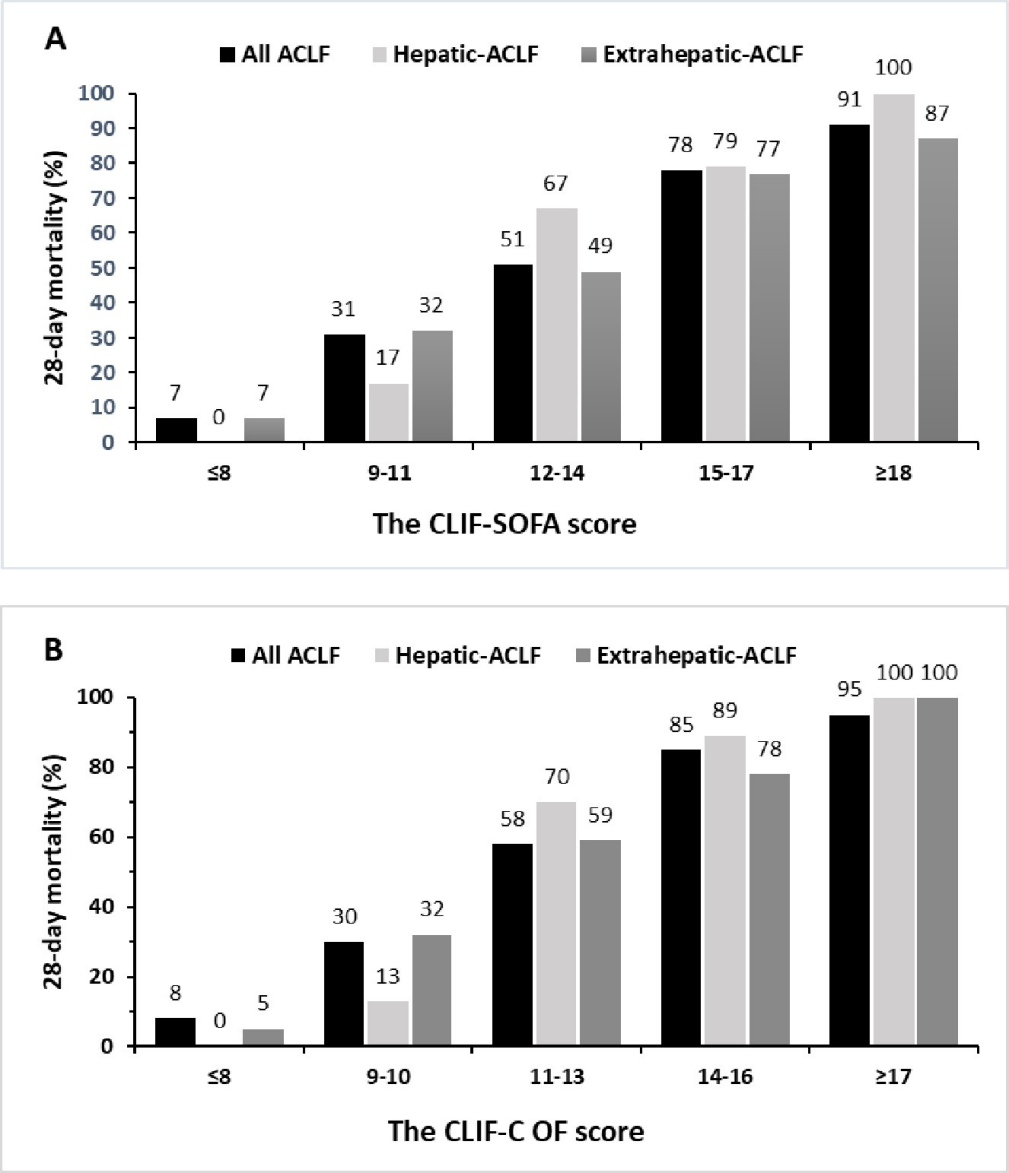
Mortality rate at 28 days according to the CLIF-SOFA and CLIF-C OF scores. Observed 28-day mortality rates of ACLF patients according to the approximate quintiles of the CLIF-SOFA (A) and CLIF-C OF (B) scores in relation to the types of acute insults.

We explored different cut-off values of CLIF-SOFA potentially useful to discriminate between the subgroups of patients at the lowest and highest risk of dying within 28 days (Fig 2A). A CLIF-SOFA of 8 or lower had a 93.0% NPV and 97.8% sensitivity, while a score of 18 or higher allowed for a 90.8% PPV and 96.3% specificity. In the 57 patients (16.6%) with a CLIF-SOFA of 8 or lower, the 28-day mortality rate (7.0%, 95% CI: 2.8%–16.7%) was 7.5 times lower than in the whole series of ACLF patients. On the other hand, a group of 65 patients (19%) with a score of 18 or higher (90.8%, 95% CI: 81.3%–95.7%) presented a 1.7-fold increase as compared to the overall mortality rate.

In this validation study, comparable results of CLIF-C OF for the prediction of 28-day mortality were observed (Fig 2B): a CLIF-C OF of 8 or lower had a 92.0% NPV and 97.8% sensitivity, while a score of 17 or higher allowed for a 95.0% PPV and 99.4% specificity. In the 50 patients (15%) with a CLIF-C OF of 8 or lower, 28-day mortality rate (8.0%, 95% CI: 3.2%–18.8%) was 6.6 times lower than in the whole series of ACLF patients. In contrast, a group of 20 patients (5.8%) with a score of 17 or higher (95%, 95% CI: 76.4%–99.1%) presented a 1.8-fold increase as compared to the overall mortality rate.

## Discussion

The present study provides external validation of scoring systems constructed on routine clinical and laboratory parameters for determining prognostic information in ACLF patients. In our cohort, the CLIF-SOFA score had the best discriminative ability in comparison with the other prognostic scores (CLIF-C ACLF, CTP, MELD, MELD-Na, iMELD, and APACHE II). The CLIF-C OF was relatively comparable with CLIF-SOFA in predicting mortality. The analysis also verified that the CLIF-SOFA and CLIF-C OF scores could be utilized in ACLF patients precipitated by different types of acute insults.

Among the several definitions for the diagnostic criteria of ACLF, the EASL-CLIF Consortium proposal has gained considerable acceptance. This diagnostic proposal defines ACLF in patients with an acute deterioration of pre-existing chronic liver disease based on the type and number of organ failures and stratifies ACLF patients according to their 28-day mortality [6]. By using this classification, we observed a high prevalence of ACLF (49%) with an association of higher ACLF grade and poor prognosis. This observation was in agreement with earlier reports of high mortality rates of ACLF grade 3 in nontransplant patients [6, 11, 17–19]. Although the pattern of acute decompensation of our cohort resembles that of decompensated cirrhosis, the present study included exclusively patients who had the rapid development of hepatic decompensation leading to organ failure within 4 weeks. This is distinct from progressive cirrhosis, which typically manifests a stepwise deterioration of liver function and eventual decompensation. In contrast to decompensated cirrhosis that carries a median survival of ~2 years, [20] our patients have high short-term mortality exceeding 50%.

It should be noted that the composition of ACLF patients in the present study was different from those in Western countries [6, 18, 19], particularly the etiologies of underlying liver disease. Our patients with ACLF typically had viral hepatitis-related cirrhosis and cryptogenic cirrhosis. In contrast to the recent data from other Asian countries, [17, 21] only 5% of the patients in this study were attributable to alcohol liver disease. This distribution may be in part due to the referral bias, and it is not representative of patients hospitalized with hepatic decompensation in Thailand [22].

ACLF usually develops following a precipitating event including either direct liver injuries or various kinds of extrahepatic insults. Similar to the CANONIC study [6] and some Asian reports [17, 21], bacterial infection constituted the predominant precipitating event of ACLF rather than reactivation of hepatitis B or superimposed viral hepatitis in our cohort. A plausible explanation could be that only cirrhotic patients were enrolled in these studies to unify chronic liver disease background, and it is well recognized that cirrhotic patients are at high risk of developing bacterial infection [1, 2]. The prognosis of ACLF depends on the severity of underlying chronic liver disease and the severity of the acute insult. There are several efforts to identify whether the nature of the acute insult apart from its severity is essential in determining the outcome of patients. A recent national cohort study from the United States highlighted that acute hepatic insult was predictive of short-term mortality for patients with ACLF [23]. A Chinese study showed comparable 28-day mortality between the hepatic and extrahepatic-ACLF groups but increased 90-day and 1-year mortality in the extrahepatic-ACLF group than hepatic-ACLF group [24]. In contrast, our study found that the short-term prognosis of hepatic-ACLF group was worse than that of extrahepatic-ACLF group, but the intermediate and long-term prognosis was similar between both hepatic and extrahepatic-ACLF groups. The difference in short-term prognosis could be explained by the fact that the majority of hepatic-ACLF patients in the Chinese study was due to flare-up or exacerbation of chronic hepatitis B, which was effectively treated with antivirals, unlike severe alcoholic hepatitis, which was the most common cause of hepatic-ACLF in our study.

The hepatic-ACLF group comprised a high number of critically ill patients resembled by a high MELD score and a large percentage of patients categorized as ACLF grades 2 and 3. Hepatic-ACLF patients were characterized by a high occurrence of liver and coagulation failures. This manifestation is synonymous to acute liver failure where an acute hepatic insult leads to rapid deterioration of liver functions. Subsequently, almost all of patients with hepatic-ACLF developed extrahepatic organ failure. This finding suggests that organs other than liver are also injured early in the course of liver dysfunction of hepatic-ACLF patients.

Given that transplantation is a marker of imminent death, analysis for the prognostic value of acute insult needs to take into account the competing nature between transplantation and death. Hence, the cumulative incidence function was used to calculate unbiased estimates for the mortality risk. The analysis revealed that the hepatic-ACLF group was not associated with increased risk of dying from any cause or undergoing liver transplantation over the 1-year period as compared to the extrahepatic-ACLF group. Given the grave prognosis of both hepatic and extrahepatic-ACLF groups, it does not seem reasonable for including patients with only hepatic insults as the diagnostic criteria for ACLF provided by the Asian Pacific Association for the Study of the Liver [25].

Identification of patients at high risk of death would help substantially in prioritizing patients for early intensive therapy, transfer to specialty units, and list for transplantation. Our analysis identified the occurrence of cerebral, respiratory, and circulatory failures during admission, and severe liver dysfunction defined by the baseline parameter of prolonged INR, high bilirubin, and low albumin levels as important factors in determining the prognosis of ACLF patients. The results confirm earlier findings that ACLF patients with more organs in failure are associated with worse outcome [6, 11, 17–19].

As expected, all of the disease severity indexes measured at ACLF diagnosis were independent predictors of death. Conventional scoring systems, such as the CTP and MELD scores and their variants have been used to define prognosis in cirrhosis and determine priorities in allocating patients for transplantation [5]. However, their prognostic accuracy is limited in ACLF due to a failure to incorporate two central prognostic determinants, namely extrahepatic organ failures and measures of systemic inflammation [26], which strengthen the pathophysiological basis of ACLF. Thus, many liver-specific scores were formulated by addressing these variables to improve the prediction of death in the setting of acutely decompensated cirrhosis with organ failure. The data obtained from patients in the CANONIC study showed the superiority of the CLIF-SOFA, CLIF-C OF, and CLIF-C ACLF scores over the CTP, MELD, and MELD-Na scores in predicting short-term and long-term mortality of patients with ACLF [6, 10]. However, these specific prognostic scores were developed from the European population, and some components of the scoring systems could be influenced by a difference in the nature of the liver disease. Here, we performed an external validation of the disease severity index and found that a prognostic accuracy of the CLIF-SOFA was relatively similar to the CLIF-C OF but significantly higher than the CLIF-C ACLF, CTP, MELD, MELD-Na, iMELD, and APACHE II among the entire cohort over the 1-year follow-up. Excellent prognostic performance of the CLIF-SOFA and CLIF-C OF likely reflects multi-organ dysfunction, which is predominant in patients with ACLF. Of interest, an excessive inflammatory response, a key player in the pathogenesis of alcoholic hepatitis was shown to be an independent factor associated with death in a Western study of ACLF patients primarily with alcoholic etiology [6]. Unfortunately, the analysis of our cohort with a few cases with alcoholic cirrhosis showed no association between the inflammatory reaction, as estimated by the leukocyte count and the mortality risk. Hence, it is not surprising that the addition of leukocyte count to the organ failure score, the performance of the CLIF-C ACLF did not be better than its predecessor, the CLIF-SOFA and CLIF-C OF in our cohort.

Based on the pivotal effects of the types of acute insults on short-term prognosis, we explored the prognostic performance of the scoring systems for the hepatic and extrahepatic groups. In both groups, the predictive accuracy of the CLIF-SOFA was relatively identical to the CLIF-C OF but significantly better than other prognostic models. Hence, the stratification of ACLF patients with the CLIF-SOFA or CLIF-C OF would be helpful for the optimization of treatment in this population. We further assessed whether the CLIF-SOFA and CLIF-C OF would allow early identification of patients at low and high risk for death. The probability of death within 28 days could be confidently excluded with an NPV of >90% in patients with a CLIF-SOFA or CLIF-C OF of ≤8. The chance of death within 28 days was a very high (PPV of >90%) in patients with a CLIF-SOFA of ≥18 or CLIF-C OF of ≥17. ACLF patients with high probabilities of death should be managed aggressively with organ support therapy in the intensive care unit and be evaluated urgently to prioritize for organ allocation.

Our study has some limitations. First, we evaluated the prognostic scores at the onset of ACLF. Some studies have shown that in patients with ACLF, sequential assessment of organ failure performs better than a single time point model [10, 19]. However, there was not enough follow-up data to compute the scores for sequential use in the present study. Second, the management of each patient was likely different, as patient care was not protocolized. Differences in the decision to initiate or withhold early organ-specific support interventions, such as renal replacement therapy that was initiated in only 9.4% of our cohort may also lead to different outcomes when compared with other centers [18, 19, 24].

In conclusion, the CLIF-SOFA and simpler CLIF-C OF are reliable measures of mortality risk in ACLF patients with a diversity of underlying liver diseases and precipitants. Both validated models could be used to stratify the risk of death and improve management of ACLF patients.

## Supporting information

S1 Fig Results of the analysis of the prognostic scoring systems for acute-on-chronic liver failure. Results of comparison of the area under the receiver operating characteristic curve of scoring systems for predicting mortality at each time point in acute-on-chronic liver failure.
(PDF)

S1 File Supporting methods and results. (XLSX)

S2 File Supporting methods. (ODT)
