# Supplementary material for: Validation of the prognostic models in acute-on-chronic liver failure precipitated by hepatic and extrahepatic insults
Source: PLoS One. 2019 Jul 10;14(7):e0219516. doi: 10.1371/journal.pone.0219516 (PMC6619802; doi:10.1371/journal.pone.0219516)
Supplement: S1 Fig — Results of comparison of the area under the receiver operating characteristic curve of scoring systems for predicting mortality at each time point in acute-on-chronic liver failure. (PDF) [file pone.0219516.s001.pdf]

This is the S1 Fig 1A. Comparison of the area under the receiver operating characteristic curve of scoring systems for predicting 28-day mortality.

	AUROC difference with CLIF-SOFA (95% CI)	P-value vs. CLIF-SOFA
**CLIF-C OF**	0.013 (-0.004 to 0.030)	0.134
**CLIF-C ACLF**	0.054 (0.016 to 0.091)	0.007
**CTP**	0.144 (0.090 to 0.199)	<0.001
**MELD**	0.207 (0.149 to 0.265)	<0.001
**MELD-Na**	0.212 (0.153 to 0.271)	<0.001
**iMELD**	0.110 (0.059 to 0.161)	<0.001
**APACHE II**	0.153 (0.091 to 0.215)	<0.001

This is the S1 Fig 1B. Comparison of the area under the receiver operating characteristic curve of scoring systems for predicting 90-day mortality.

	AUROC difference with CLIF-SOFA	P-value vs. CLIF-SOFA
**CLIF-C OF**	0.025 (0.006 to 0.044)	0.010
**CLIF-C ACLF**	0.050 (0.011 to 0.088)	0.013
**CTP**	0.176 (0.119 to 0.233)	<0.001
**MELD**	0.249 (0.191 to 0.306)	<0.001
**MELD-Na**	0.250 (0.192 to 0.307)	<0.001
**iMELD**	0.138 (0.087 to 0.189)	<0.001
**APACHE II**	0.189 (0.126 to 0.251)	<0.001

This is the S1 Fig 1C. Comparison of the area under the receiver operating characteristic curve of scoring systems for predicting 6-month mortality.

	AUROC difference with CLIF-SOFA	P-value vs. CLIF-SOFA
**CLIF-C OF**	0.020 (-0.001 to 0.040)	0.064
**CLIF-C ACLF**	0.032 (-0.009 to 0.073)	0.137
**CTP**	0.162 (0.102 to 0.221)	<0.001
**MELD**	0.240 (0.181 to 0.300)	<0.001
**MELD-Na**	0.240 (0.180 to 0.300)	<0.001
**iMELD**	0.124 (0.070 to 0.177)	<0.001
**APACHE II**	0.163 (0.098 to 0.229)	<0.001

This is the S1 Fig 1D. Comparison of the area under the receiver operating characteristic curve of scoring systems for predicting 1-year mortality.

	AUROC difference with CLIF-SOFA	P-value vs. CLIF-SOFA
**CLIF-C OF**	0.026 (0.005 to 0.046)	0.018
**CLIF-C ACLF**	0.034 (-0.007 to 0.076)	0.111
**CTP**	0.172 (0.112 to 0.232)	<0.001
**MELD**	0.241 (0.181 to 0.301)	<0.001
**MELD-Na**	0.242 (0.182 to 0.301)	<0.001
**iMELD**	0.123 (0.069 to 0.177)	<0.001
**APACHE II**	0.167 (0.100 to 0.234)	<0.001
