# Supplementary material for: Validation of the prognostic models in acute-on-chronic liver failure precipitated by hepatic and extrahepatic insults
Source: PLoS One. 2019 Jul 10;14(7):e0219516. doi: 10.1371/journal.pone.0219516 (PMC6619802; doi:10.1371/journal.pone.0219516)
Supplement: Supplementary file 2 [file pone.0219516.s003.odt]

Patient Records for the study entitled “the validation of prognostic score for ACLF”

Demographic Data

_ _ _				Patient Number

_ _				Age

_ 				Gender (1= male, 2= female)

__ 				Causes of cirrhosis (1= HBV, 2=HCV, 3=Alcohol, 4=AIH, 5=PBC, 6=NAFLD, 7=cryptogenic, 8=Hemochromatosis, 9= Wilson disease, 10=PSC, 11= other _______)

Clinical Details on Admission

_ _ _ 		 		Systolic BP (mmHg)

_ _ _ 		 		Diastolic BP (mmHg)

_ _ _				Heart rate (beat/min)

_ _ ._				Temperature (Celsius)

__ __ __		Body weight (kg) 

_ _ _				Height (cm)

__   				Ascites on admission (0= No, 1= Yes)

__   				Jaundice on admission (0= No, 1= Yes)

__                           Child–Pugh Score        

__ __ __ gm/day 	Alcohol intake 

__ 				Smoking (0 = Nonsmoker, 1= Exsmoker, 2= current smoker) 

__ 				Diabetes (0= No, 1= Yes) 

__ 				Hypercholesterolemia (0= No, 1= Yes) 

__ 				Hypertensive (0= No, 1= Yes) 

					If yes, treatment_______________________________________ 

__ 				Ischemic Heart Disease (0=No,1= Yes) 

__ 				Cerebrovascular Disease (0=No,1= Yes) 

__ 				Chronic renal failure (0=No,1=Yes) 

__ 				COPD (0=No,1=Yes)

__   				Hematologic disease (0=No,1=Yes)

__					if yes type____________________ treatment _______________

__   				Previous intraabdominal surgery (0= No,1=Yes___________________)

__ 				Malignancy (0= No,1= Yes) 

__					if yes 1= Bowel, 2= Lung, 3=Hematological, 4=prostate, 						5=breast, 6= skin, 7=other___________) 

     __ __ day               Time from first previous liver decompensation to admission 

__				History of cirrhotic complication (0= No,1= Yes)

__		             		If yes: 1= variceal bleeding, 2= SBP, 3=ascites, 4=Hepatic 

						Encephalopathy, 5= Other__________)    

__                           Grade of Hepatic Encephalopathy (grade 0-4)

__   				Previous SBP (0= No,1= Yes)

__   				Hx of variceal bleeding (0= No, 1= Yes)

__   				Hx of variceal band ligation (0= No, 1= Yes)

_ 				Precipitating event (0= unknown, 1= hepatic, 2= extrahepatic) 

						Etiology_____________

Laboratory Data on admission

__ . __ mg/dL 		Total bilirubin 

__ __ __ U/L			AST 

__ __ __ U/L 		ALT 

__ __ __ __ U/L		Alkaline Phosphatase 

__ __ __ __ U/L		GGT 

__. __ g/dL			Albumin 

__. __ g/dL			Globulin 

__ __ . __ sec. 		Prothrombin time 

__.__                                  International Normalization Ratio

__ __ __ mg/dL 		Creatinine

__ __ __ mg/dL 		Plasma glucose 

__ __ __ mg/dL 		Total Cholesterol 

__ __ __ __ mg/dL 	Triglyceride 

__ __ __ mg/dL 		HDL cholesterol 

__ __. __ g/dL 		Hemoglobin

__ __ __ __ __ /mL	Leukocyte count

__ __ __ __ __ /mL	Neutrophil count

__ __ __ x 109/L 		Platelet count

__ __ __ mmol/l 		Sodium

__ __ __ mmol/l 		Potassium

__ __ __ mmol/l 		Chloride

__ __ __ mmol/l 		Bicarbonate

__ __					MELD score

Laboratory Data at anytime 

__ 					Hepatitis B serology 1= sAb +ve, 2= sAg +ve, 3= sAg –ve. 

__ 					Hepatitis C 1= Ab –ve, 2= Ab or RNA +ve

__ __ .__ mg/dL		Ceruloplasmin

__ __ __ __ mcg/L 	Ferritin 

__ __ % 			Transferrin saturation 

__ : __ __ __ __ 		Anti-nuclear Ab

__ : __ __ __ 			Anti-smooth muscle Ab

__ : __ __ __ 			Anti-mitochondrial Ab

Clinical Data and Laboratory of CLIF-SOFA during Admission

__   				Admission with new ascites or worsening of ascites (0= No, 1= Yes)

__                           Admission with Hepatic Encephalopathy (grade 0-4)

__   				Admission with SBP (0= No,1= Yes; organism________________)

__   				Admission with Bacterial infection (0= No,1= Yes; 

						organ______________organism________________)

__   				Admission with variceal bleeding (0= No, 1= Yes)

__					Organ failure (0=no, 1=yes)

__					Number of organ failure (0-6)

__					Time of organ failure (0=no, 1=on admission, 2=after admission)

__					Day after admission for developing organ failure	

__                                       Grade of Hepatic Encephalopathy (0-4)

_ _ _ 		 			Systolic BP (mmHg)

_ _ _ 		 			Diastolic BP (mmHg)

     _ _ _ 					Mean arterial pressure (mmHg)

__                                       Inotropic Drug (0=No, 1=yes)

    __ __ %                              Oxygen saturation (SpO2)

    __ __                                   Respiratory support (0=No, 1=Yes)

    __					    If Yes; type of device: 1=O2 cannular, 2=O2 mask with bag,

                                               		3= ET tube with Mechanical Ventilator)

__                                       FiO2 of respiratory support device

__ __ mmHg                      PaO2 If have arterial blood gas

__ . __ mg/dL 		Total bilirubin 

__ __ __ U/L			AST 

__ __ __ U/L 		ALT 

__ __ __ __ U/L		Alkaline Phosphatase 

__. __ g/dL			Albumin 

__. __ g/dL			Globulin 

__ __ . __ sec. 		Prothrombin time 

__.__                                  International Normalization Ratio

__ __ __ mg/dL 		Creatinine

__ __ __ mg/dL 		Serum glucose 

__ __. __ g/dL 		Hemoglobin

__ __ __ x 109/L		Leukocyte count

__ __ __ x 109/L 		Platelet count

__ __ __ x 109/L		Ascitic PMN count

__ .__				SAAG

__				       	Hemoculture (0=No, 1=yes; organism_______________)

__				    	Ascitic culture (0=No, 1=yes; organism_______________)

__				    	Urine culture (0=No, 1=yes; organism_______________)

__				    	Sputum culture (0=No, 1=yes; organism_______________)

Management for ACLF

__   			             Intensive Care Unit 0=No, 1=yes

__ __		 		    Day after admission to ICU (day)

__ __		 		    Day in ICU (day)	

__                               Use of inotropic Drug (0=No, 1=yes)

__                               Abdominal paracentesis (0=No, 1=yes; amount ascites release ____L)

__                               Use of intravenous albumin (0=No, 1=yes)

__                               PRC transfusion (0=No, 1=yes_____ unit)

__                               Use of antibiotic Drug (0=No, 1=yes__________________)

__                               Endoscopic Therapy for GI hemorrhage (0=No, 1=yes)

__					if yes (1=PU, 2=EV, 3=GV, 4=Other__________)	

__                               Mechanical Ventilator (0=No, 1=yes)

__                               Renal Replacement Therapy (0=No, 1=yes)

__                               Hepatic Dialysis (0=No, 1=yes)

__                               Liver Transplantation (0=No, 1=yes)

_ _ /_ _ /_ _ 			if yes, date of OLT (DD/MM/YY)

Follow-up Data after first admission

__ __ / __ __/ __ __ __ __ 	Date of last follow-up (MM/DD/YYYY) 

__ 				Death during the first 30 days after admission (0= no, 1= yes)

__ 				Death during the first 90 days after admission (0= no, 1= yes)

__ 				Death 0= no, 1= yes) 

__ __ / __ __ / __ __ __ __ 	Date of Death 

__ 				Cause of death 1= liver failure, 2= variceal bleed, 3= HCC, 

				4= other malignancy, 5= IHD, 6= CVD, 7= Infection, 8= other, 9= don’t know 

_________________		Other 

__ 				New Ascites or worsening of ascites (0= no, 1= yes) 

__ __/ __ __/ __ __ __ __ 	Date of diagnosis of ascites 

__ 				Hepatic encephalopathy (0= no, 1= yes) 

__ __/ __ __/ __ __ __ __ 	Date of diagnosis of encephalopathy 

__ 				Variceal hemorrhage (0= no, 1= yes) 

__ __ / __ __/ __ __ __ __ 	Date of variceal hemorrhage 

__ 				Jaundice (0= no, 1= yes) 

__ __ / __ __/ __ __ __ __ 	Date of diagnosis of jaundice 

__ 				Hepatocellular carcinoma (0= no, 1= yes) 

__ __ / __ __/ __ __ __ __ 	Date of diagnosis of hepatocellular carcinoma 

__ 				Liver transplantation (0= no, 1= yes) 

__ __ / __ __/ __ __ __ __ 	Date of liver transplantation
